# The Cause of Death in the Colic of Horses

**Published:** 1882-01

**Authors:** 


THE CAUSE OF DEATH IN THE COLIC OF HORSES.—Dr. Immanuel Munk, of the Berlin Veterinary School, has made some suggestive experiments regarding the above subject. They are recorded in full in the supplemental volume of the *Archivs für Physiologie* for 1880.

It occurred to him that, in the colic to which horses are subject, the cause of death might be, in part at least, due to the absorption of carbolic acid and other products of fermentation in the intestinal canal.

He first made a series of experiments in order to determine the amount of carbolic acid excreted off daily in the urine. Eight healthy horses were taken and fed upon a fixed ration of hay, 4.5 kilos. (20 lbs.); oats, 2.5 kilos. (12 lbs.). The daily average of drinking water was 10 litres (11 quarts). We give the table of the averages of the eight experiments:

Average weight of horses, 406 kilos. (850 lbs.); average daily amount of water drank, in litres, 10.3; average daily amount of urine, in litres, 3.01; average total amount of tribomphenol, 10.886 grains (ʒ ii$\frac{3}{4}$).

These experiments showed that a horse weighing 800 lbs. passed daily an average of 10.9 grains (ʒ ii.$\frac{3}{4}$) of tribromphenol and 3 grains

(℥¾) of phenol, making a total of about ℥iiss. of impure carbolic acid a day. Three of the animals were killed at the end of the experiments, and their intestines examined, but no carbolic acid found in them. On the other hand, the urine of horses that died of colic was examined and found to contain less than one-half the normal amount of the acid in question.

The intestines of the same animals were also examined, but no acid found. There were, however, chemical substances from which it might easily be developed.

Some further experiments were made to test the lethal dose of carbolic acid. In the case of dogs, a dose of four grains to the pound weight would produce sure and certain death.

We can not by any means consider that Dr. Munk has proved carbolic acid poisoning to be the cause of death in colic. Still, it may be one of the factors, and one that should be borne in mind, since this agent is a heart-poison.

